# HDP2: a ribosomal DNA (NTS-ETS) sequence as a target for species-specific molecular diagnosis of intestinal taeniasis in humans

**DOI:** 10.1186/s13071-018-2646-6

**Published:** 2018-02-27

**Authors:** María D. Flores, Luis M. Gonzalez, Carolina Hurtado, Yamileth Monje Motta, Cristina Domínguez-Hidalgo, Francisco Jesús Merino, María J. Perteguer, Teresa Gárate

**Affiliations:** 1 Parasitology Department, Centro Nacional de Microbiología, Instituto de Salud Carlos III, Crtra, Majadahonda-Pozuelo, km 2.2, 28220 Majadahonda, Madrid Spain; 2 Ciencias Farmacéuticas y de la Salud, Facultad de Farmacia, Universidad San Pablo-CEU, 28668 Montepríncipe, Madrid Spain; 3 Programa de Medicina, Facultad de Salud, Universidad del Magdalena, 47004 Santa Marta D.T.C.H, Colombia; 4 Microbiología, Hospital Universitario Severo Ochoa, 28911 Leganés, Madrid Spain

**Keywords:** Taeniasis, *Taenia solium*, *Taenia saginata*, Diagnosis, cPCR, qPCR, Ribosomal DNA

## Abstract

**Background:**

*Taenia solium*, *T. asiatica* and *T. saginata* tapeworms cause human taeniasis and are the origin of porcine and bovine cysticercosis. Furthermore, *T. solium* eggs can cause human cysticercosis, with neurocysticercosis being the most serious form of the disease. These helminth infections are neglected tropical diseases and are endemic in several countries in the Americas, Asia and Africa. As a result of globalization, migration in particular, the infections have been extending to non-endemic territories. Species-specific diagnosis of taeniasis is subject to drawbacks that could be resolved using molecular approaches. In the present study, conventional and real-time amplification protocols (cPCR and qPCR) based on the *T. saginata* HDP2 sequence were applied in the differential diagnosis of taeniasis (*T. saginata*, *T. solium*) in both fecal samples and proglottids expelled by patients. The HDP2 homolog in *T. solium* was cloned and characterized.

**Results:**

Semi-nested cPCR and qPCR (Sn-HDP2 cPCR and Sn-HDP2 qPCR) amplified *T. saginata* and *T. solium* DNA, with an analytical sensitivity of 40 and 400 fg, respectively, and identically in both protocols. Eighteen taeniasis patients were diagnosed directly with *T. saginata* or *T. solium*, either from proglottids or fecal samples with/without eggs (detected using microscopy), based on the optimized Sn-HDP2 qPCR. After cloning, the *T. solium* HDP2 homolog sequence was confirmed to be a ribosomal sequence. The HDP2 fragment corresponded to a non-transcribed sequence/external transcribed repeat (NTS/ETS) of ribosomal DNA. Compared with the *T. saginata* HDP2 homolog, the *T solium* HDP2 sequence lacked the first 900 nt at the 5′ end and showed nucleotide substitutions and small deletions.

**Conclusions:**

Sn-HDP2 cPCR and Sn-HDP2 qPCR were set up for the diagnosis of human taeniasis, using proglottids and fecal samples from affected patients. The new Sn-HDP2 qPCR protocol was the best option, as it directly differentiated *T. saginata* from *T. solium*. The diagnosis of an imported *T. solium*-taeniasis case and nine European *T. saginata* cases was relevant. Finally, the cloning and sequencing of the *T. solium* HDP2 fragment confirmed that HDP2 was part of a ribosomal unit.

**Electronic supplementary material:**

The online version of this article (10.1186/s13071-018-2646-6) contains supplementary material, which is available to authorized users.

## Background

*Taenia solium*, *T. asiatica* and *T. saginata* tapeworms cause human taeniasis and are the origin of porcine and bovine cysticercosis [1]. In addition, *T. solium* eggs can cause cysticercosis in humans, with neurocysticercosis being the most serious form of the disease [2]. These helminth infections are neglected tropical diseases and are endemic in several countries in the Americas, Asia and Africa [2, 3]. As a result of globalization, human migration in particular, taeniasis and cysticercosis have been extending to other regions where they had been eliminated during the 20th century [4].

The impact of taeniasis/cysticercosis on health and socio-economic development in endemic regions is significant enough that several initiatives have been undertaken to control them [5, 6]. Sensitive and specific diagnostic tools are therefore of considerable importance in aiding these initiatives [7]. Species-specific identification of *Taenia* spp. is a fundamental component of the diagnosis of taeniasis, and suitable treatment and prevention of the transmission of cysticercosis play a key role, as human *T. solium* carriers are the origin of cysticercosis. In this regard, molecular techniques are excellent alternatives that complement immunodiagnosis and traditional parasitological methods, which are affected by specificity (e.g. *Taenia* coproantigen ELISA) and specificity/sensitivity (e.g. *Taenia* eggs and microscopy) [1]. The supporting information (Additional file 1: Table S1; Additional file 2: Table S2) summarizes the molecular targets used in the identification of *Taenia* spp. The targets include mitochondrial and ribosomal DNA, repetitive DNA sequences and/or genes encoding relevant antigens. Based upon these sequences, we investigated the *T. saginata* HDP2 repetitive sequence [8]*,* which hybridized differentially with DNA from *T. saginata* (3 ng) and *T. solium* (25 ng) [8]. The *T. saginata* HDP2 fragment was sequenced and further characterized [9], and it was found that the 5′ end of HDP2 (5PHDP2, approximately 1000 bp) was not present in the homologous sequence of *T. solium.* The information extracted was used to design cPCRs, which were in turn used for species-specific identification of the taeniid [9, 10], and for the detection of *T. solium* DNA in cerebrospinal fluid from neurocysticercosis patients [11]. Next, several *T. saginata* and *T. asiatica* isolates were analyzed, and it was confirmed that the HDP2 fragment was polymorphic owing to a variable representation of partially repeated sequences, rather than to nucleotide sequence divergence, thus proving its relevance as a diagnostic target [12, 13]. Prior to this study, the PCRs derived from HDP2 had not been applied to samples from taeniasis patients. In addition, the homolog of HDP2 in *T. solium* had not been characterized.

In the present study, both Sn-HDP2 cPCR and Sn-HDP2 qPCR were performed based on the *T. saginata* HDP2 sequence, and their analytical sensitivities were determined using purified *T. saginata* and *T. solium* genomic DNA (gDNA). These protocols were applied to carry out the species-specific diagnosis of taeniasis in both fecal samples and proglottids expelled by infected humans. Furthermore, the HDP2 homologous sequence from *T. solium* was cloned and characterized.

## Methods

### Parasite material

A frozen *T. solium* adult was provided by Professor Elizabeth Ferrer, Universidad de Carabobo, Valencia, Venezuela. *Taenia solium* cysticerci were a gift from Professor Edda L. Sciutto, Instituto de Investigaciones Biomédicas, Universidad Nacional Autónoma de México, México DF, México. A *T. saginata* adult was supplied by Dr Sabino Puente Puente, Hospital La Paz-Carlos III, Madrid, Spain. The parasitic material was stored at -80 °C until use; genomic DNA (gDNA) from *T. saginata* and *T. solium* samples was isolated as described elsewhere [14].

### Clinical samples

The study samples comprised 8 fecal samples and 16 proglottids (fragmented or non-preserved, unidentifiable by morphology) from 18 individuals with taeniasis (Table 1). In the case of stool, 1–3 serial samples per patient were used. The clinical samples belonged to collection C.0003989, which is registered at the ISCIII Biobank, and were managed following current ethical recommendations, according to Spanish Royal Decree 1716/2011 about the requirements of biobanks in biomedical research.

**Table 1:** Species-specific diagnosis of human taeniosis using Sn-HDP2 cPCRs and Sn-HDP2 qPCR. In serial fecal samples, the comparison of both PCR and microcopy observation (MO) results is included

Case	Origin	Sex	Age (yrs)	Species	Proglottids		Fecal samples					
					Sn HDP2 cPCRa	Sn HDP2 qPCR	Sn HDP2 cPCRa & qPCR			MO		
							1	3	3	1	2	3
1	Spain	Female	18	*T. saginata*	+	+						
2	France	Male	47	*T. saginata*	+	+						
3	Spain	Female	34	*T. saginata*	+	+	+	+		–	–	
4	Spain	Male	33	*T. saginata*	+	+						
5	Spain	Male	37	*T. saginata*	+	+						
6	Spain	Female	29	*T. saginata*	+	+						
7	Spain	Female	32	*T. saginata*			+			+		
8	Spain	Female	33	*T. saginata*	+	+	+			+		
9	Spain	Female	84	*T. saginata*	+	+						
10	Paraguay	Female	48	*T. saginata*	+	+	+	+	+	+	–	–
11	Bolivia	Female	29	*T. saginata*	+	+						
12	Nicaragua	Female	37	*T. saginata*	+	+						
13	Colombia	Female	43	*T. solium*	+	+						
14	Ethiopia	Female	65	*T. saginata*			+			+		
15	Unknown	Male	35	*T. saginata*	+	+						
16	Unknown	Female	34	*T. saginata*	+	+						
17	Unknown	Male	15	*T. saginata*	+	+						
18	Unknown	Male	47	*T. saginata*	+	+						

^a^Sn-HDP2 cPCR amplicons were sequenced

*Key*: + positive; − negative

Prior to DNA extraction, 1 g of each stool sample was suspended in 8 ml of saline solution and concentrated using Bioparapred-Midicolumns (Leti Diagnostics, Barcelona, Spain). The supernatants were then discarded, and 200 mg of the pellets was used for total DNA extraction [15]. DNA from serial fecal samples and proglottids was purified using QIAamp DNA extraction kits (QIAamp Fast DNA Stool Mini Kit and QIAamp DNA Mini Kit, respectively) (Qiagen, Venlo, the Netherlands), following the manufacturer’s recommendations. DNA samples were eluted in 200 μl of water.

### Microscopy analysis

Clinical samples were analyzed by optical microscopy according to standard protocols [16].

### Amplification protocols (PCR): Sn-HDP2 cPCR and Sn-HDP2 qPCR

The Sn-HDP2 cPCR used F2,2 (5′-CTT CTC AAT TCT AGT CGC TGT GGT CAG-3′) and R10 (5′-GAG GAA TAG ATG GAT GAA GGG-3′) as primers for the first amplification and F2,2 and R1m (5′-GAC GAA GAA TGG AGT TGA AGG-3′) as primers for the second amplification. The cPCR was performed as previously described [11]. The first cPCR reaction was performed using 40 μl of fecal DNA, or 15 μl of proglottid DNA as template. Negative and positive controls were performed for each assay. For the second amplification, 2 μl of a 1:500 dilution in water from the first amplification was used. Amplicons were sequenced, as described below.

The primers used in Sn-HDP2 qPCR were the same as for Sn-HDP2 cPCR. The first reaction was performed in a total volume of 20 μl, containing 2 μl of 10× LightCycler® FastStart DNA Master SYBR Green I (Roche Diagnostics, Barcelona, Spain). In addition, 1 μl of each primer at 10 μM, DNA template (15 μl fecal DNA plus 1 μl of sterile water and 5 μl of DNA template from proglottids plus 11 μl of sterile water). Negative and positive controls were performed for each assay. The thermocycler conditions were as follows: 95 °C for 10 min; 40 cycles of 95 °C for 10 s, 56 °C for 15 s, 72 °C for 20 s; 72 °C to 99 °C (continuous acquisition). For the second amplification, the reaction was carried out in a total volume of 20 μl using the same reagents as for the first qPCR and 2 μl of a 1:500 dilution in water from the first reaction. The thermocycler conditions of the second amplification were as follows: 95 °C for 10 min; 35 cycles of 95 °C for 10 s, 58 °C for 15 s, 72 °C for 20 s; 72 °C to 99 °C (continuous acquisition). Amplicons were sequenced, and melt curves were estimated.

### Analytical sensitivity of Sn-HDP2 cPCR and Sn-HDP2 qPCR

Serial dilutions (1:10) from purified *T. saginata* and *T. solium* gDNA prepared from 1 ng/μl to 1 fg/μl were amplified using the two amplification protocols.

### Construction of a *T. solium* genomic library

gDNA from *T. solium* cysticerci was used to prepare a parasite DNA library with Lambda Fix II vector following a previously described protocol [14].

### Cloning of the *T. solium* HDP2 sequence

Two strategies were used to clone the *T. solium* HDP2 sequence: (i) *T. solium* gDNA library screening with the *T. saginata* HDP2 homolog probe [9, 13] and (ii) cPCR-cloning using *T. solium* gDNA as a template and ribosomal DNA-specific primers derived from *T. solium* 28S and 18S ribosomal DNA sequences. The supporting information (Additional file 3: Table S3) includes the characteristics of both universal and walking primers used to complete the sequencing of *T. solium* HDP2 subclones, which were obtained by library screening. In addition, cPCR ribosomal primers and walking primers (Additional file 3: Table S3) were used to directly clone and sequence the *T. solium* HDP2 amplification product. The DNA from recombinant plasmids was prepared using standard protocols. Sequencing, sequence data alignments and other analyses were performed as previously described [14, 17, 18].

## Results and discussion

Sn-HDP2 cPCR and Sn-HDP2 qPCR amplified *T. saginata* and *T. solium* DNA with an analytical sensitivity of 40 fg and 400 fg, respectively, which was maintained in both protocols. In the case of *T. saginata* DNA in particular, sensitivity was comparable to that reported for other molecular markers [19–21].

**Fig. 1:**
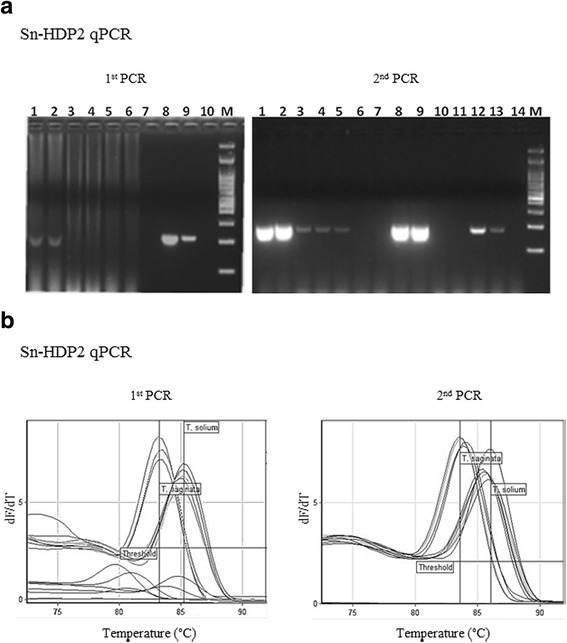
Diagnosis of human taeniosis using Sn-HDP2 cPCR and Sn-HDP2 qPCR. **a** Sn-HDP2 cPCR applied to 3× fecal samples from a patient with taeniasis. Amplification products fractionated on 2% agarose gels and stained by GelRed. First PCR: Lanes 1, 2: fecal sample 1 (positive by microscopy); Lanes 3, 4: fecal sample 2; Lanes 5, 6: fecal sample 3 (negative by microscopy); Lane 8: *T. saginata* DNA (positive control); Lane 9: *T. solium* DNA (positive control); Lane 10: no DNA (negative control); Lane M: 100 bp DNA Ladder (NIPPON Genetics Europe, Dueren, Germany). Second PCR: Lanes 1, 2: fecal sample 1; Lanes 3, 4: fecal sample 2; Lanes 5, 6: fecal sample 3; Lanes 8, 9: *T. saginata* DNA (positive control); Lanes 12, 13: *T. solium* DNA (positive control); Lane 14: no DNA (negative control); Lane M: 100 bp DNA Ladder (Genetics). Sn-HDP2 cPCR amplification products were sequenced. **b** Melting curves corresponding to the new Sn-HDP2 qPCR (both first and second PCR runs), applied to both *T. saginata* and *T. solium* DNA

Sn-HDP2 cPCR had previously been successfully used for the diagnosis of neurocysticercosis based on cerebrospinal fluid samples from infected Mexican patients [11]. At that time, a species-specific amplification protocol was not needed, as *T. solium* larva is the only taeniid that can invade the human central nervous system. However, in the case of taeniasis, which is produced by both taeniids, Sn-HDP2 cPCR yielded an amplicon with an identical size for both *T. saginata* and *T. solium* DNA [9, 10]; sequencing of the amplification product was essential for the specific identification of the taeniid [11, 12]. A new diagnostic amplification protocol was set up to circumvent the sequencing step by taking advantage of the differences in HDP2 in nucleotides between the two species. The novel Sn-HDP2 qPCR showed a distinct melting temperature (Tm) according to the *Taenia* species analyzed (Tm of 83.30 °C for *T. saginata* and 86.00 °C for *T. solium*), and the difference in Tm had already been detected in the first amplification (Fig. 1b). Furthermore, for proglottids, the first reaction usually yielded conclusive diagnostic results in both amplification protocols, as was the case in fecal samples when the parasite material was sufficient. In previous years, other HDP2 cPCRs protocols were applied for identification of *T. saginata* in fecal samples artificially spiked with known numbers of *T. saginata* eggs [22]. Moreover, as indicated above, amplifications of several molecular targets such as mitochondrial genes (*cox*1, *nad*5, *nad*1, 12S), and ribosomal ITS1 region and 28S and 5.8S genes [19, 21, 23] were described in various amplification protocols (cPCR, qPCR, LAMP) using mono- or multiplex systems, with very good results for the identification of *Taenia* spp. [19–21, 23, 24]. In comparison with Triplex Taq-Man (T3qPCR) [21], Sn-HDP2 qPCR did not need a labeled probe, although it did not include an internal PCR control and was not checked with *T. asiatica* DNA.

In order to confirm the applicability of Sn-HDP2 cPCR and Sn-HDP2 qPCR in the diagnosis of infected human samples, proglottids and fecal samples from taeniasis patients were examined using both PCR assays. The results obtained are summarized in Table 1. With proglottids, the two PCRs were able to diagnose taeniasis in the first amplification as follows: *T. saginata* in patients from Europe (*n* = 9), South America (*n* = 3), Africa (*n* = 1) and from undetermined regions (*n* = 4), with ages ranging from 15 to 84 years; *T. solium* in a 43 year-old Latin American individual. In the case of fecal samples, the PCRs yielded positive results, and *T. saginata* was identified, even in stools negative for taeniid eggs (Case 3 and Case 10, Table 1). The identification was confirmed by agarose gel electrophoresis of the cPCR amplification product (Fig. 1a) followed by amplicon sequencing. Molecular approaches have already been used in human samples for the diagnosis of taeniasis in endemic regions, with excellent specificity and sensitivity [20, 21, 23]. The molecular identification of a *T. solium* tapeworm carrier in the present study is noteworthy, given the epidemiological consequences of the transmission of cysticercosis [25] and the possible need for surveillance measures in countries where the parasite has been eliminated. Today, migration from taeniid-endemic regions could extend transmission [26]. In addition, the frequent diagnosis of *T. saginata* in the taeniasis patients studied, in accordance with recent data from Europe [27], suggests the need for better reporting of taeniasis/bovine cysticercosis if the disease is to be better controlled [25].

**Fig. 2:**
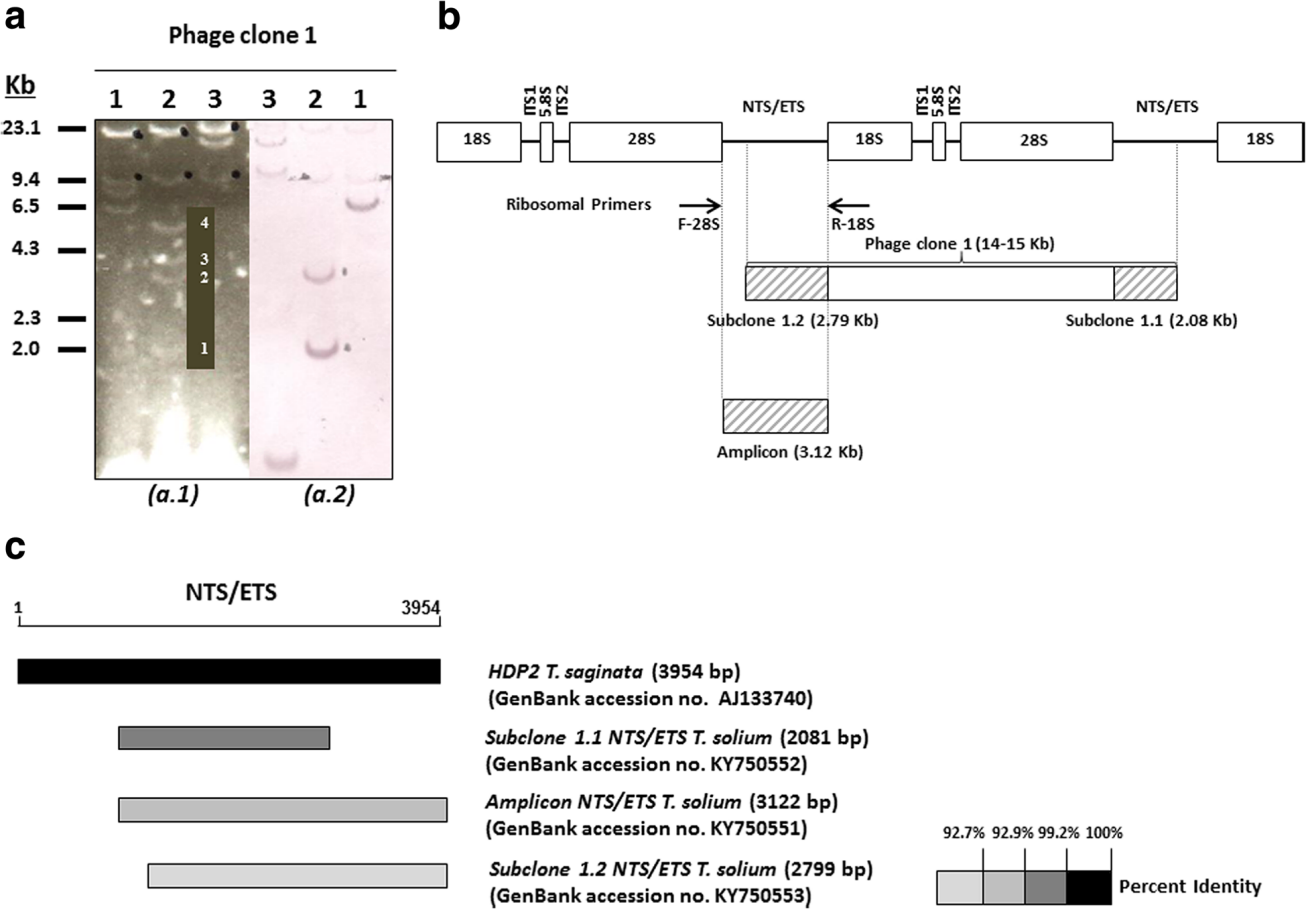
DNA sequence organization and similarities of the *T. solium* HDP2 fragment. **a** *a.1*: Restriction fragment patterns for *T. solium* recombinant phage clone 1 DNA digested by *Sac*I (Lane 1); *Sal*I (Lane 2); *Xba*I (Lane 3); *a.2*: Southern blot: *T. saginata* HDP2 DNA sequence digoxigenin-11-dUTP-labeled hybridization with *T. solium* recombinant phage clone 1 DNA digested by *Sac*I (Lane 1); *Sal*I (Lane 2); *Xba*I (Lane 3). **b** Diagram showing the genomic organization of the *T. solium* HDP2 subclones 1.1 and 1.2 and the *T. solium* HDP2 amplicon, with respect to the structure of the ribosomal DNA repeats (-18S-ITS1-5.8S-ITS2-28S-NTS/ETS-). **c** Physical alignment of *Taenia* HDP2 (NTS/ETS) fragments. The NTS/ETS ribosomal repeat is represented by a line, with the *T. saginata* HDP2 unit as the reference sequence. The boxes in grayscale correspond to the percent homology with the *T. saginata* HDP2 fragment (black box represents 100% identity). *T. solium* subclones 1.1 and 1.2 and the ribosomal amplicon are represented by boxes in different shades of gray according to similarities

In-depth analysis and characterization of the properties of the HDP2 homolog of *T. solium* were performed through cloning of the DNA sequence by screening a *T. solium* gDNA library with the *T. saginata* HDP2 homolog [14] and through direct cPCR-cloning using *T. solium* gDNA and specific ribosomal DNA primers [28]. A positive recombinant phage (phage clone #1, *c.*14–15 kb) and a specific genomic amplicon (*c.*3.0 kb) were obtained (Fig. 2). To determine which region of the phage fragment corresponded to the HDP2 sequence, phage clone #1 DNA was digested by the *Sal*I restriction enzyme. The digestion yielded four fragments (Fig. 2a: a.1), two of which were hybridized with the *T. saginata* HDP2 sequence probe, namely, subclones #1.1 and #1.2 (Fig. 2a: a.2). *T. solium* phage subclones #1.1 and #1.2 were 2081 bp and 2799 bp long, respectively (Fig. 2b), and showed high similarities in their DNA sequences (GenBank: subclone #1.1: KY750552; subclone #1.2: KY750553). They also exhibited a high similarity with the DNA sequence of the *T. solium* ribosomal amplicon (3122 bp) cloned by cPCR (Fig. 2c), with few nucleotide substitutions and small deletions (GenBank: amplicon: KY750551). The DNA similarities discovered and the BLAST analysis data confirmed that *T. solium* HDP2 (3.12 kb) was a ribosomal gene, as was previously suggested for other taeniid homologous HDP2 molecules [13], when significant similarities between *T. saginata* HDP2 and the repetitive ribosomal sequences Taiwan *Taenia* pTTr 3.1, *T. saginata* pTSgr 3.1 and pTSgr 2.4, were demonstrated [29]. The *T. solium* fragment (3.12 kb) and subclones #1.1 and #1.2, in comparison with the *T. saginata* HDP2, lacked the first 900 nt at the 5′ end, as described elsewhere [9], and showed nucleotide substitutions and small deletions. Specifically, the largest deletion was located in *T. solium* HDP2 clones (from 590 to 620 nt).

The *T. solium* HDP2 structure within the ribosomal tandem repeats was further studied by restriction enzyme mapping based on the characteristics of both phage subclones and amplicon sequences. Thus, the rough size of cloned phage 1 was 14–15 kb. Together with the characteristics of standard rRNA genes determined by BLAST analysis, this phage included two partial copies of the ribosomal amplicon HDP2 (3.12 kb) at its 5′ and 3′ ends and the phage subclones #1.2 (2.79 kb) and #1.1 (2.08 kb), which were located in two different and contiguous non-transcribed sequence/external transcribed repeat (NTS/ETS) regions (Fig. 2b). Since information on the structural organization of rDNA sequences in *Taenia* species is poor, these data make it possible to conclude that the phage 1 sequence corresponded to a *T. solium* ribosomal DNA repeat plus an NTS/ETS region (Fig. 2b), with the HDP2 fragment being an NTS/ETS unit, according to the description made for the sequence and structure of the *Caenorhabditis elegans* rDNA repeat [30]. Therefore, the genomic variations detected between the characteristics of HDP2 in both taeniids could explain the following: (i) the different hybridization profiles found when the *T. saginata* and *T. solium* gDNAs were hybridized with the labeled *T. saginata* HDP2 DNA sequence [8, 9]; (ii) the non-amplification of *T. solium* DNA by the R1F1-HDP2-PCR [9]; and (iii) the differential sensitivity of taeniids determined by the amplification protocols described in the present paper.

Finally, the repetitive nature of HDP2 sequences (ribosomal NTS/ETS units) and the differences in nucleotide composition between *Taenia* species would explain the diagnostic properties of the molecule as a target in high-sensitivity, species-specific amplification protocols.

## Conclusions

In the present study, Sn-HDP2 cPCR and Sn-HDP2 qPCR based on proglottids and fecal samples were applied to diagnose taeniasis in humans, even though the fecal sample did not contain microscopy-proven parasitic forms and the proglottid was damaged. Sn-HDP2 qPCR proved to be the better diagnostic option. The finding of a *T. solium* tapeworm carrier and the eight cases of *T. saginata* was relevant, considering the crucial epidemiological consequences of transmission of cysticercosis. The cloning and sequencing of the HDP2 homolog in the *T. solium* genome confirmed that the DNA was ribosomal, specifically an NTS/ETS unit, and explained the differences in performance of the amplification protocols according to the specific composition of this genomic region in *T. solium* and *T. saginata*.

## Additional files

Additional file 1: Table S1. DNA markers and molecular protocols used to identify the taeniid species involved in human taeniasis. (PDF 35 kb)

Additional file 2: Table S2. References included in Additional file 1: Table S1. (PDF 18 kb)

Additional file 3: Table S3. Universal and walking primers used for *T. solium* HDP2 sequencing. (PDF 60 kb)

## Abbreviations

cPCR: Conventional polymerase chain reaction
ETS: External transcribed spacer
LAMP: Loop-mediated isothermal amplification
NTS: Non-transcribed spacer
qPCR: Real-time polymerase chain reaction
WHO: World Health Organization
