# Supplementary material for: HDP2: a ribosomal DNA (NTS-ETS) sequence as a target for species-specific molecular diagnosis of intestinal taeniasis in humans
Source: Parasit Vectors. 2018 Feb 27;11:117. doi: 10.1186/s13071-018-2646-6 (PMC6389257; doi:10.1186/s13071-018-2646-6)
Supplement: Supplementary file 1 — DNA markers and molecular protocols used to identify the taeniid species involved in human taeniasis. (PDF 35 kb) [file 13071_2018_2646_MOESM1_ESM.pdf]

Additional file 1: Table S1. DNA markers and molecular protocols used to identify the taeniid species involved in human taeniasis.

DNA type	DNA marker	Molecular protocols (DNA Hybridization, RFLP, PCRs, Sequencing, LAMP...)	Samples/ Sensitivity (Detection limit for gDNA, sensitivity for taeniasis carriers diagnosis) and Specifity	Species Identification	Reference
rDNA	pTS10	DNA Hybridization	gDNA: 100 ng	T.sol	[1,2]
nDNA	HDP1: highly repetive<br>HDP2: repetitive	DNA Hybridization	HDP1, gDNA: 3 ng<br><br>HDP2, gDNA: 3 ng/25 ng	HDP1: T.sag<br><br>HDP2: T.sag/T.sol	[3]
rDNA	pTTr 3.1 and pTSgr 3.1, pTSgr 2.4	PCR	gDNA: 500 ng	TaiwanT-T.sag	[4]
rDNA, mtDNA	28S rRNA , ITS1-rRNA, CO1	Sequencing and PCR/RFLP	_	T.sol-T.sag-Asian Taenia	[5]
nDNA, mtDNA	Highly repetitive DNA (pTSol9, pTsag-16)	DNA Hybridization	gDNA: 50-100 ng; eggs: 1-50,<br><br>gDNA: 100-150 ng	pTSol9: T.sol<br><br>pTsag-16: Tsag	[6]
mtDNA	12S rDNA	PCR/Sequencing	200-800 ng	T.sol-T.sag and other taeniids	[7]
nDNA	HDP1, HDP2	HDP1 PCR<br>Mx-HDP2 PCR	gDNA: < 10 pg<br>gDNA: 10 pg	HDP1: T.sag<br>HDP2: T.sag-T.sol	[8,9]
rDNA	5.8S and ITSs rRNA	PCR-REA (PCR/RFLP)	gDNA: 100 ng	T.sol-T.sag	[10]
mtDNA	CO1 and CYTb	PCR/Seq	_	T.sol genotypes	[11,12]
mtDNA	CO1 and CYTb	(PCR-BESS T Base analysis)	_	T.sag-T.asia-T.sol genotypes	[13]
mtDNA	12S rDNA	PCR/RFLP	_	T.sag-T.sol	[14]
mtDNA	CO1	PCR/RFLP	--	T.sag-T.asia-T.sol genotypes	[15]
nDNA	HDP2	Mx-HDP2 PCR<br>Sn-HDP2 PCR	Feces: 4375 eggs/2g stool sediment<br>137 eggs /2g stool sediment	T.sag	[16]

Additional file 1: Table S1. DNA markers and molecular protocols used to identify the taeniid species involved in human taeniasis.

nDNA	HDP2	HDP2 PCR/RFLP<br>Mx-HDP2 PCR	gDNA: 10 ng	T.sag-T.asia	[17]
mtDNA	CO1	Multiplex/ nested PCR	Feces: 50 eggs/g feces	T.sag-T.asia-T.sol genotypes	[18]
mtDNA	CO1	PCR/RFLP	Feces: 34 eggs/2g stool sediment	T.sag-T.sol	[19]
nDNA	Tso31	Nested PCR	Feces: 10 eggs/250 mg (40 eggs/g)<br>gDNA: 100 fg<br>Fecal samples from taemiasis carriers: 97% sensitivity; 100% specificity	T.sol	[20]
mtDNA	Valine tRNA and NADH	Multiplex PCR	Feces:10 eggs	T.sag-T.asia -T.sol	[21]
mtDNA, nDNA	CO1, CLP (cathepsin L-like cysteine peptidase)	LAMP (Loop-mediated isothermal amplification)<br>LAMP/RFLP	1 copy of the genes<br>Feces: 5eggs/g	T.sol-T.sag-T.asia	[22]
nDNA	HDP2	Multiplex PCR	gDNA:5-10 ng	T.sol-T.sag-T.asia	[23]
nDNA	Ag2	PCR/Sequencing	gDNA: 100 ng	T.sol genotypes	[24]
rDNA	ITS1/FAM and Quasar probes	Real time PCR	gDNA:,T sol > 1 fg;<br>T.sag > 2.5 fg,<br>Fecal samples from taemiasis carriers: 82.7% sensitivity<br>99.0% specificity	T.sag-T.sol	[25]
mt DNA	CO1	Pyrosequencing	Proglottid, scolex	T.sag-T.asia -T.sol	[26]
mt DNA	12S, Nad5, Cox1, Nad1	PCR/sequencing	Cyst fluids, CSF, proglottids.<br>Detection limit:<br>T.sag (Cox1, 1 pg; 12S, 100fg);<br>E.gra (Nad5, 100 pg; 12S, 10 pg;)	*Taenia* sp and *Echinococcus* sp diagnosis and typing	[27]

Additional file 1: Table S1. DNA markers and molecular protocols used to identify the taeniid species involved in human taeniasis.

			Specificity: cross-amplifications		
mit RNA	small (rrnS) and large (rrnL) subunits	Dual PCR	1 pg of *Echinococcus* gDNA	*Taenia* sp, *Echinococcus* sp, Diagnosis and typing	[28]
rDNA<br><br>mitDNA	ITS1(T.sol)/Cy5 probe<br>CO1(T.sag; T.asia)/HEX;FAM probes	Triplex Taq-Man (T3qPCR)	--	ITS1: T.sol<br><br>CO1: T.sag-T.asia	[29]
