# Supplementary material for: HDP2: a ribosomal DNA (NTS-ETS) sequence as a target for species-specific molecular diagnosis of intestinal taeniasis in humans
Source: Parasit Vectors. 2018 Feb 27;11:117. doi: 10.1186/s13071-018-2646-6 (PMC6389257; doi:10.1186/s13071-018-2646-6)
Supplement: Supplementary file 2 — References included in Additional file 1: Table S1. (PDF 18 kb) [file 13071_2018_2646_MOESM2_ESM.pdf]

Additional file 2: Table S2. References included in Additional file 1: Table S1.

1. Rishi AK, McManus DP. DNA probes which unambiguously distinguish *Taenia solium* from *T. saginata.* Lancet. 1987;2:1275-6.
2. Rishi AK, McManus DP. Molecular cloning of *Taenia solium* genomic DNA and characterization of taeniid cestodes by DNA analysis. Parasitology. 1988;97:161-76.
3. Harrison LJ, Delgado J, Parkhouse RM. Differential diagnosis of *Taenia saginata* and *Taenia solium* with DNA probes. Parasitology. 1990;100:459-61.
4. Zarlenga DS, McManus DP, Fan PC, Cross JH. Characterization and detection of a newly described Asian taeniid using cloned ribosomal DNA fragments and sequence amplification by the polymerase chain reaction. Exp Parasitol. 1991;72:174-83.
5. Bowles J, McManus DP. Genetic characterization of the Asian *Taenia,* a newly described taeniid cestode of humans. Am J Trop Med Hyg. 1994;50:33-44.
6. Chapman A, Vallejo V, Mossie KG, Ortiz D, Agabian N, Flisser A. Isolation and characterization of species-specific DNA probes from *Taenia solium* and *Taenia saginata* and their use in an egg detection assay. J Clin Microbiol. 1995;33:1283-8.
7. von Nickisch-Rosenegk M, Silva-Gonzalez R, Lucius R. Modification of universal 12S rDNA primers for specific amplification of contaminated *Taenia* spp. (Cestoda) gDNA enabling phylogenetic studies. Parasitol Res. 1999;85:819-25.
8. González LM, Montero E, Harrison LJ, Parkhouse RM, Garate T. Differential diagnosis of *Taenia saginata* and *Taenia solium* infection by PCR. J Clin Microbiol. 2000;38:737-44.
9. González LM, Montero E, Sciutto E, Harrison LJ, Parkhouse RM, Garate T. Differential diagnosis of *Taenia saginata* and *Taenia solium* infections: from DNA probes to polymerase chain reaction. Trans R Soc Trop Med Hyg. 2002;96 Suppl. 1:S243-S250.
10. Mayta H, Talley A, Gilman RH, Jimenez J, Verastegui M, Ruiz M, Garcia HH, Gonzalez AE. Differentiating *Taenia solium* and *Taenia saginata* infections by simple hematoxylin-eosin staining and PCR-restriction enzyme analysis. J Clin Microbiol. 2000;38:133-7.
11. Okamoto M, Nakao M, Sako Y, Ito A. Molecular variation of *Taenia solium* in the world. Southeast Asian J Trop Med Public Health. 2001;32 Suppl 2:90-3.
12. Nakao M, Okamoto M, Sako Y, Yamasaki H, Nakaya K, Ito A. A phylogenetic hypothesis for the distribution of two genotypes of the pig tapeworm *Taenia solium* worldwide. Parasitology. 2002;124:657-62.
13. Yamasaki H, Nakao M, Sako Y, Nakaya K, Sato MO, Mamuti W, Okamoto M, Ito A. DNA differential diagnosis of human taeniid cestodes by base excision sequence scanning thymine-base reader analysis with mitochondrial genes. J Clin Microbiol. 2002;40:3818-21.
14. Rodriguez-Hidalgo R, Geysen D, Benítez-Ortiz W, Geerts S, Brandt J. Comparison of conventional techniques to differentiate between *Taenia solium* and *Taenia saginata* and an improved polymerase chain reaction-restriction fragment length polymorphism assay using a mitochondrial 12S rDNA fragment. J Parasitol. 2002;88:1007-11.
15. Margono SS, Ito A, Sato MO, Okamoto M, Subahar R, Yamasaki H, Hamid A, Wandra T, Purba WH, Nakaya K, Ito M, Craig PS, Suroso T. *Taenia solium* taeniasis/cysticercosis in Papua, Indonesia in 2001: detection of human worm carriers. J Helminthol. 2003;77:39-42.

16. Nunes CM, Lima LG, Manoel CS, Pereira RN, Nakano MM, Garcia JF. *Taenia saginata*: polymerase chain reaction for taeniasis diagnosis in human fecal samples. Exp Parasitol. 2003;104:67-9.
17. González LM, Montero E, Morakote N, Puente S, Díaz De Tuesta JL, Serra T, López-Velez R, McManus DP, Harrison LJ, Parkhouse RM, Gárate T. Differential diagnosis of *Taenia saginata* and *Taenia saginata asiatica* taeniasis through PCR. Diagn Microbiol Infect Dis. 2004;49:183-8.
18. Yamasaki H, Allan JC, Sato MO, Nakao M, Sako Y, Nakaya K, Qiu D, Mamuti W, Craig PS, Ito A. DNA differential diagnosis of taeniasis and cysticercosis by multiplex PCR. J Clin Microbiol. 2004;42:548-53.
19. Nunes CM, Dias AK, Dias FE, Aoki SM, de Paula HB, Lima LG, Garcia JF. *Taenia saginata*: differential diagnosis of human taeniasis by polymerase chain reaction-restriction fragment length polymorphism assay. Exp Parasitol. 2005;110:412-5.
20. Mayta H, Gilman RH, Prendergast E, Castillo JP, Tinoco YO, Garcia HH, Gonzalez AE, Sterling CR; Cysticercosis Working Group in Peru. Nested PCR for specific diagnosis of *Taenia solium* taeniasis. J Clin Microbiol. 2008;46:286-89.
21. Jeon HK, Chai JY, Kong Y, Waikagul J, Insisiengmay B, Rim HJ, Eom KS. Differential diagnosis of *Taenia asiatica* using multiplex PCR. Exp Parasitol. 2009;121:151-56.
22. Nkouawa A, Sako Y, Nakao M, Nakaya K, Ito A. Loop-mediated isothermal amplification method for differentiation and rapid detection of *Taenia* species. J Clin Microbiol. 2009;47:168-74.
23. González LM, Bailo B, Ferrer E, García MD, Harrison LJ, Parkhouse MR, McManus DP, Gárate T. Characterization of the *Taenia* spp HDP2 sequence and development of a novel PCR-based assay for discrimination of *Taenia saginata* from *Taenia asiatica*. Parasit Vectors. 2010;3:51.
24. Sato MO, Sako Y, Nakao M, Wandra T, Nakaya K, Yanagida T, Ito A. A possible nuclear DNA marker to differentiate the two geographic genotypes of *Taenia solium* tapeworms. Parasitol Int. 2011;60:108-10.
25. Praet N, Verweij JJ, Mwape KE, Phiri IK, Muma JB, Zulu G, van Lieshout L, Rodriguez-Hidalgo R, Benitez-Ortiz W, Dorny P, Gabriël S. Bayesian modelling to estimate the test characteristics of coprology, coproantigen ELISA and a novel real-time PCR for the diagnosis of taeniasis. Trop Med Int Health. 2013;18:608-14.
26. Thanchomnang T, Tantrawatpan C, Intapan PM, Sanpool O, Janwan P, Lulitanond V, Tourtip S, Yamasaki H, Maleewong W. Rapid molecular identification of human taeniid cestodes by pyrosequencing approach. PLoS One. 2014;9:e100611.
27. Roelfsema JH, Nozari N, Pinelli E, Kortbeek LM. Novel PCRs for differential diagnosis of cestodes. Exp Parasitol. 2016;161:20-6.
28. Boubaker G, Marinova I, Gori F, Hizem A, Müller N, Casulli A, Jerez Puebla LE, Babba H, Gottstein B, Spiliotis M. A dual PCR-based sequencing approach for the identification and discrimination of *Echinococcus* and *Taenia* taxa. Mol Cell Probes. 2016;30:211-7.
29. Ng-Nguyen D, Stevenson MA, Dorny P, Gabriël S, Vo TV, Nguyen VT, Phan TV, Hii SF, Traub RJ. Comparison of a new multiplex real-time PCR with the Kato Katz thick smear and copro-antigen ELISA for the detection and differentiation of *Taenia* spp. in human stools. PLoS Negl Trop Dis. 2017;11:e0005743.
