# Supplementary material for: HDP2: a ribosomal DNA (NTS-ETS) sequence as a target for species-specific molecular diagnosis of intestinal taeniasis in humans
Source: Parasit Vectors. 2018 Feb 27;11:117. doi: 10.1186/s13071-018-2646-6 (PMC6389257; doi:10.1186/s13071-018-2646-6)
Supplement: Supplementary file 3 — Universal and walking primers used for T. solium HDP2 sequencing. (PDF 60 kb) [file 13071_2018_2646_MOESM3_ESM.pdf]

Additional file 3: Table S3. Universal and walking primers used for *T. solium* HDP2 sequencing.

**Name**	**Sequence (5’- 3’)**
pBluescript KS	TCGAGGTCGACGGTATC
pBluescript SK	GATCCACTAGTTCTAGAGCG
T3	AATTAACCCTCACTAAAGGG
T7	TAATACGACTCACTATAGGG
SP6	TATTTAGGTGACACTATAG
1.1F	ACATTCCAAAGTGATAAATGAATAGCAAACAGTTTTTTGC
1.1R	TTAAAGAGAAATTAGTGACTATTAAGTGGATACCAAAAGGGAGGT
1.1WP1F	CGAGTGCGTGCACATAAAGCATGT
1.1WP1R	ATGTATTCCACCCCCCGTACTATG
1.2F	GTCCTCTGCTGGGCAGAAAAGAAA
1.2R	ATGTATTCCACCCCCCGTACTATG
WP1.2F	AGCATGTAGGCTACGCTGTTCGTA
WP1.2R	GTCGAAGCAAGAAGTGCAAGAAGTGTTTG
6.1F	GAACACGAGGGCGAGGGAGAT
6.1WP2	GTTCGAACAAACAAAGTATTACACAGCCCCTC
28SA	AGCCCAGCGCCGAAGCCTGCGGC
28SB	CTAGAGTCGGGGTGTTTGTGAATGC
28SC	CTGTCTATCTGACCTCGGATCAGT
18SA	GCCAGCTAGATGAGGCGCTGAAGAAGGAGG
18SB	GTGAGCAGGAAACACTTTCTCATTA
18SC	GAGCAGGATAAAATCCATCGAAGTCGGATCCA
